# Intravenous thrombolysis and mechanical recanalization for acute ischemic stroke in deep brain stimulation patients: a case series

**DOI:** 10.1007/s00415-023-11765-4

**Published:** 2023-05-22

**Authors:** Johannes Meyne, Mirjam Domschikowski, Johannes Hensler, Ann-Kristin Helmers, Daniela Berg, Günther Deuschl, Steffen Paschen

**Affiliations:** 1 Department of Neurology, University Hospital Schleswig-Holstein, Campus Kiel and Kiel University, Arnold-Heller-Str. 3, 24105 Kiel, Germany; 2 Department of Radiology and Neuroradiology, University Hospital Schleswig-Holstein, Campus Kiel and Kiel University, Kiel, Germany; 3 Department of Neurosurgery, University Hospital Schleswig-Holstein, Campus Kiel and Kiel University, Kiel, Germany

**Keywords:** Deep brain stimulation, Intravenous thrombolysis, Endovascular mechanical recanalization therapy

## Abstract

**Background:**

Intravenous thrombolysis (IVT) and endovascular mechanical thrombectomy therapy (MT) are well established in the treatment of acute ischemic stroke. It is currently unclear whether these treatments can be applied in patients with previous deep brain stimulation (DBS) surgery and how long the interval to the DBS operation should be.

**Methods:**

Four patients with ischemic stroke and IVT or MT were included in this retrospective case series. Data on demographics, genesis, severity and course of the stroke and the DBS indication were extracted and evaluated. Furthermore, a literature review was conducted. Outcomes and hemorrhagic complications after IVT, MT or intra-arterial thrombolysis in patients with prior deep brain stimulation surgery and intracranial surgery were analyzed.

**Results:**

Four patients with acute ischaemic stroke and previous DBS surgery were treated with IVT (2 patients), MT (1) or a combined therapy of IVT and MT (1). The time interval to the previous DBS surgery was between 6 and 135 months. In these four patients, no bleeding complications occurred. The literature review revealed four publications with a total of 18 patients, who were treated with IVT, MT or intra-arterial thrombolysis. Of these 18 patients, only 1 had undergone deep brain stimulation surgery, the other 17 patients had received brain surgery for other reasons. Bleeding complications occurred in four of the 18 reported patients, but not in the DBS case. All four patients with bleeding complications were reported to have died as a result. In three of the four patients with fatal outcome, surgery was less than 90 days before the onset of stroke.

**Conclusion:**

IVT and MT were tolerated by four patients with ischemic stroke more than 6 months after DBS surgery without bleeding complications.

## Introduction

Recanalization therapies, either endovascular mechanical recanalization therapy (MT) or intravenous thrombolysis (IVT) are well established in the treatment of acute ischemic stroke. In patients with previous surgery, the timing and location of surgery are relevant when weighing benefits and the risk of bleeding, especially for the indication of IVT [3]. Currently, it is unclear whether IVT and/or MT can be administered in patients who had undergone previous deep brain stimulation (DBS) surgery and how long the interval to a subsequent revascularization therapy should be. The brain tissue around the electrodes could be more vulnerable with an increased risk of bleeding, comparable to the increased risk of hematoma at the surgical site for other operations [13]. In addition, the rigid electrode and the connecting cable are chronically left in the brain moving simultaneously with all motions of the head. In a case report of a 40-year-old woman (2.25 years after implantation of DBS electrodes for epilepsy), IVT was performed without complications [6].

In the current American heart association/American stroke organization (AHA/ASA) guidelines, intravenous alteplase therapy is considered potentially harmful for patients with intracranial surgery within the previous 3 months (evidence class III) [11]. However, the safe interval between IVT and previous brain surgery is discussed controversially [5].

In the following, we present four case reports of patients with prior DBS surgery who received IVT and/or MT for cerebral ischemic stroke. In addition, the existing literature on IVT/MT in ischemic stroke and previous intracranial surgery was comprehensively reviewed.

## Methods

Four patients with DBS and subsequent ischemic stroke and IVT/MT were identified from the records of the University hospital Schleswig–Holstein, Campus Kiel. Demographic data, stroke risk factors, time of symptom onset, stroke severity using the National Institutes of Health Stroke Scale (NIHSS), time of angiographic treatment, complications, presumed cause of stroke, cause of death, and functional outcome follow-up using the modified Rankin Scale (mRS) were extracted.

For each CT scan at admission, the Alberta Stroke Program Early CT Score (ASPECTS) and, if a CT-angiography and -perfusion was performed, the localization of a large vessel occlusion was determined [2]. Recanalization was defined as Thrombolysis in Cerebral Infarction (TICI) score 2b-3 [7]. Hemorrhagic transformation in subsequent CT scans was classified as follows: no signs of hemorrhagic transformation, hemorrhagic infarction, parenchymal hemorrhage, and symptomatic intracranial hemorrhage [9]. All CT scans were assessed by a blinded rater (JH).

### Methods of literature review

For review of the literature regarding outcome and hemorrhagic complications after use of IVT, MT or intra-arterial thrombolysis (IAT) in patients with prior deep brain stimulation or intracranial surgery, we searched MEDLINE and Google Scholar from 1995 to April 2022 (search date: 29 April 2023). Search terms were first (tissue plasminogen activator OR tPA OR fibrinolysis OR tenecteplase OR alteplase OR thrombolysis OR (thrombolytic therapy)) AND ((deep brain stimulation) OR (neurostimulation)), and second (stroke AND (off label) AND (((mechanical recanalisation) OR (mechanical recanalization) OR (mechanical thrombectomy) OR (endovascular therapy)) OR (thrombolysis OR (thrombolytic therapy)))). Only reports published in English were included. All potentially relevant articles were screened as full text by two authors (JM and SP) and references of selected studies were reviewed to retrieve additional relevant citations.

Data on study design, number of included patients, type of intracranial surgery, time elapsed since surgery, type of treatment (IVT/IAT), time window of stroke onset till start of treatment, complications and functional outcome were extracted and evaluated.

#### Ethics board approval

The study was approved by the local ethics committee (D497/22; final approval in June 2022).

#### Data availability

Data not provided in the article because of space limitations may be shared (anonymized) at the request of any qualified investigator for purposes of replicating procedures and results.

## Results

Four patients with IVT and/or MT and previous DBS operation were included in the analysis (3 female, 1 male; Table 1). DBS operations were performed between 2005 and 2019. Three of the patients underwent bilateral subthalamic nucleus (STN) DBS surgery for advanced Parkinson’s disease with motor fluctuations, and one patient underwent bilateral ventro-intermediate nucleus DBS for medical refractory essential tremor. The DBS system implanted was a Medtronic Kinetra^®^ in three and a Boston Scientific Gevia^®^ in one patient. At the time of stroke, the patients were between 68 and 77 years old, and the time interval to previous DBS surgery was between 6 and 135 months. On arrival at the emergency department, symptom severity as assessed with the NIHSS was between 7 and 24 points. Blinded evaluation of the CCT at admission yielded ASPECTS ranging from 7 to 10 points. Three of the patients had a large vessel occlusion.

**Table 1:** Demographic, treatment and outcome data of four patients treated with intravenous thrombolysis and/or mechanical recanalization with prior deep brain stimulation surgery

	Case 1	Case 2	Case 3	case 4	Range
Age at stroke onset (years)	71	68	75	77	68–77
Time elapsed since DBS surgery (months)	17	6	89	135	6–135
NIHSS at admission	14	15	7	24	7–24
Time window: stroke onset till IVT start (h)	< 4.5	Unclear	1.7	< 3	
ASPECTS	10	8	10	7	7–10
Large vessel occlusion (branch)	MCA (M2)/PCA (P2)	MCA (M1)	n. a	MCA (M2)	
Treatment: IVT/MT	IVT	MT	IVT	IVT + MT	
Dosage alteplase (mg)	70	n. d	77	63	63–77
Result of mechanical recanalization (TICI)	n. d	3	n. d	3	3
Classification of hemorrhagic transformation in subsequent CT scan [9]	No HT	No HT	No HT	HI1	
Time from IVT/MT to control CT scan (hours)	13	11.5	19	19	11.5–19
Etiology	ESUS	Atrial fibrillation	Microangiopathic	Atrial fibrillation	
Complications	No	No	No	Pneumonia, circulatory insufficiency	
mRS 3 months post-stroke	3	3	4	6	3–6
Gender (m/f)	f	m	f	f	
Diagnosis	ET	PD	PD	PD	
Onset of disease	2001	2009	1985	1999	
DBS surgery date	2008–12	2019–10	2005–12	2007–01	
Implanted DBS system	Medtronic, Kinetra®	Boston scientific, Gevia®	Medtronic, Kinetra®	Medtronic, Kinetra®	
Stimulation target	VIM	STN	STN	STN	
Stroke onset	2010–05	2020–04	2013–05	2018–04	

*MCA* middle cerebral artery, *ASPECTS* Alberta Stroke Program Early CT Score, *DBS* deep brain stimulation, *ESUS* embolic stroke of undetermined source, *ET* essential tremor, *HI1* hemorrhagic infarction with small petechiae along the margins of the infarct, *HT* hemorrhagic transformation, *IVT* intravenous thrombolysis, *M1/M2* horizontal/insular segment of the middle cerebral artery, *MT* endovascular mechanical recanalization therapy, *mRS* modified Rankin scale, *no HT* no signs of hemorrhagic transformation, *n. d.* not done, *NIHSS* National Institute of Health Stroke Scale (at admission of the patient), *P2* post-communicating segment of the posterior cerebral artery, *PCA* posterior cerebral artery, *PD* idiopathic Parkinson’s disease, *STN* subthalamic nucleus, *TICI* post-thrombectomy reperfusion status [Thrombolysis in Cerebral Infarction], *VIM* ventralis intermediate nucleus

Two patients received IVT, one patient underwent MT and one patient a combined therapy of IVT and MT. IVT was administered with recombinant tissue plasminogen activator (rtPA) alteplase using the standard weight-adjusted dose according to the manufacturer’s instructions 4.5 h after stroke onset, at latest. Both patients with MT were successfully mechanically recanalized (both TICI 3). In subsequent CT scans 11.5–19 h after IVT or MT, case 4 showed a hemorrhagic infarction with small petechiae along the margins of the infarct in subsequent CT imaging. In the other three patients, there was no evidence of hemorrhagic transformation.

The functional outcome as assessed with the mRS 3 months after stroke onset varied between 3 (moderate impairment) and 6 (death due to pneumonia and circulatory insufficiency in one of the IVT patients).

### Review of bleeding complications following IVT, IAT and MT in patients with brain surgeries

The literature search yielded 139 search results, of which 4 publications with a total of 18 cases with previous DBS surgery (1 patient) or other intracranial brain surgery (17 patients) prior to IVT or IAT were included in the analysis (Table 2). No complications occurred in the patient with DBS surgery 27 months prior to IVT [6]. Symptomatic intracranial hemorrhage was noted in three patients and small subarachnoid hemorrhage and gastric bleeding in one patient, respectively. Thus, 4 of 18 had bleeding complications, and all 4 patients were reported to have died as a result. In 3 of the 4 patients with fatal outcome, the surgery was less than 90 days before stroke onset, and in 1 of the 4 patients, it was more than 90 days ago (Table 2).

**Table 2:** Reported cases receiving IVT or IAT with previous intracranial surgery and their functional outcome

Author	Study design	*n*	Surgery	Time elapsed since surgery/trauma (days)	treatment (IVT/IAT)	Time window stroke onset till IVT/EVT start (h)	Complications	Outcome
Henninger [6]	Retrospective	1	DBS surgery	820	IVT	1.6	No	mRS 0
Voelkel [13]	Retrospective	1	ACM aneurysma coiling	73	IVT	Not reported	Small subarachnoidal hemorrhage and gastric bleeding	Death
Chalela [4] (Review: Aleu [1])	Retrospective	3	Craniotomy	< 14	IAT		SICH, n = 2	Death, *n* = 2
Meretoja [10]	Retrospective	13	CNS-pathology: a history of operation or trepanation of traumatic subdural hematoma or major cerebral contusion (8), meningioma operations (3), or normal pressure hydrocephalus shunting (2)	> 90	IVT	< 4.5	SICH, *n* = 1	Death, *n* = 1; mrs 0–1, *n* = 2; mrs 0–2, *n* = 6

*DBS* deep brain stimulation, *IAT* intra-arterial thrombolysis, *IVT* intravenous thrombolysis, *mRS* modified Ranking Scale, *SICH* symptomatic intracranial hemorrhage

## Discussion

We report four patients with previous DBS surgery who received IVT or MT after a time interval ranging from a minimum of 6 to 135 months after DBS surgery. None of the patients experienced a bleeding complication. To our knowledge, this has only been shown for one other patient so far [6].

According to the current understanding, intracranial hemorrhage due to DBS surgery is believed to be predominantly caused by insertion of the DBS electrodes [8]. In addition, in the early postoperative phase (up to 72 h after surgery), hemorrhages may be leveraged by blood pressure derailments. Treatment recommendations include restarting of oral anticoagulation or antiplatelet therapy 14–28 days after DBS surgery [12] if necessary for comorbidities, reflecting normalization of the general postoperative bleeding risk after this period.

In addition, in our literature search for patient reports with IVT and MT following previous intracranial surgery, only case series with a small total number of patients (total *n* = 18) were found (Table 2). A time interval of less than 90 days between IVT/IAT and previous brain surgery was identified as the most important risk factor for a fatal outcome: 3 of 4 bleeding complications with fatal outcome occurred within 3 months after previous intracranial surgery. Pathophysiologically, this can be explained by the increased vulnerability of brain tissue in the early phase after surgery [13]. Although the review of the literature indicates that the risk of bleeding at the previous surgical site is significantly increased and that the 3-months interval after surgery is a vulnerable period, a direct comparison between DBS surgery and surgeries such as aneurysm surgery is limited. DBS surgery results in limited brain damage along DBS trajectories, whereas aneurysm surgery may not cause any brain lesions at all. Thus, to assess the bleeding risk of IVT after previous DBS surgery, in addition to the three patients with IVT treatment in our case series, only the present case report of the patient with a time interval of 27 months since DBS surgery can be considered.

Furthermore, the risk of bleeding after previous DBS operation is most likely higher with IVT than with MT. However, data on this are not available.

Some limitations have to be considered. First, a publication bias, such as that the outcome of single patients or of patients with a particularly poor outcome, may not have been published. Second, the focus of this study is to assess the risk of IVT and MT after previous DBS surgery. The here reported small number of five cases does not allow a definite conclusion. The comparison to patients with an-other indication for intracranial brain surgery is reasonable, but the risks of IVT and MT are not directly comparable. However, we believe that considering the overall low number of published cases with brain surgery and IVT and/or MT, this approach provides the best possible approximation of risks.

With our report of the highest number of patients with IVT/MT after DBS of one center and review of the literature, we suggest that IVT and MT can be considered in patients with previous DBS surgery and the occurrence of ischemic stroke under risk–benefit considerations. The increased incidence of IVT associated surgical site bleeding within 3 months of neurosurgery may also apply for IVT after DBS surgery but so far no cases of IVT are reported within this period. As these are extremely rare events, data on such interventions should be collected in a registry before any final conclusions can be drawn.

## Conclusion

IVT and MT were tolerated by four patients with ischemic stroke more than 6 months after DBS surgery without bleeding complications. This may serve as information for physicians who need to decide about IVT and MT for their patients with DBS and ischemic stroke.

## Declarations

### Classification of evidence

This study provides class IV evidence that IVT and MT may be applied in patients with DBS and ischemic stroke based on an individual risk-benefit analysis, taking into account the time between previous DBS surgery and IVT and/or MT.”

## Glossary

DBS: Deep brain stimulation
IVT: Intravenous thrombolysis
MT: Endovascular mechanical recanalization therapy
